# Prescriptions

**Published:** 1893-03

**Authors:** 


TREATMENT OF BURNS IN CHILDREN.

The burns are first washed with solution of boric acid, and the following mixture applied on pads of absorbent gauze :—

℞ Aquæ calcis . . . . . . . . . . . 50.0 grms.
Olei lini . . . . . . . . . . . . . . 50.0 grms.
Thymoli . . . . . . . 0.05 to 0.10 grm.

—DR. WERTHEIMER, in *Pharm. Post.*

CASTOR-OIL.

A castor-oil which can scarcely be recognized as such is made as follows:—

℞ Castor-oil . . . . . . . . . . . . . 30 parts.
Bitter almond . . . . . . . . . . 2 parts.
Sugar . . . . . . . . . . . . . . . . . 30 parts.
Gum tragacanth . . . . . . . . ½ part.
Orange-flower water . . . . 10 parts.
Water . . . . . . . . . . . . . . . . 120 parts.

This is to be made into an emulsion, of which every 5 parts will be one part castor-oil.—PROFESSOR WHELPLEY, in *Bulletin of Pharmacy.*

DYSPEPSIA.

M. Jules Simon has found the following treatment very useful in cases of obstinate indigestion:—

℞ Tincture of cascarilla . . . . .
Tincture of cinnamon . . . .
Tincture of gentian . . . . . .
Tincture of colombo . . . . .
Tincture of rhubarb. . . aa . . ʒ ij.
Tincture of nux vomica . . . ʒ j.

Ten to twenty drops, in a little water, before each meal.—*Times and Register.*

HYSTERIA.

℞ Tinct. Asafœtidæ . . . . . . 2 drachms.
Tinct. Valerianæ . . . . . 2 drachms.
Aquæ Camphoræ . q. s. . 4 ounces.

M. Sig. One drachm every hour.

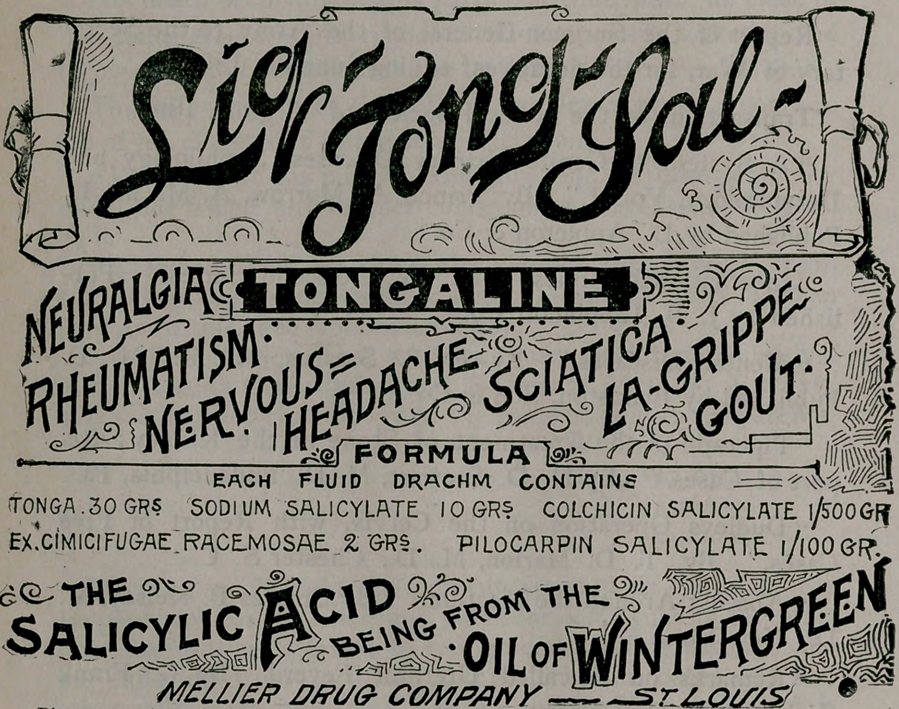

### TYPHOID FEVER.

℞ Acid, carbolic.......... ʒ ss.

Chloroform ............ ℥ j.

Gum acacia............ ʒ j.

Syrup.........q. s. ad.. ℥ iv.

M. Sig.: Teaspoonful three or four times daily. If there is a tympanitic condition of the bowels, it disappears at once.—I. J. BUSH, M. D., in *Med. Ann.*

### FOR BRONCHITIS.

℞ Ammonii Carbonat...16 grains.
Syrup. Tolu.......... 4 drachms.
Tinct. Scillæ.........40 minims.
Tinct. Cinchonæ Comp.2 drachms.
Spirit. Chloroformi... 4 minims.
Aquæ Rosæ.......... 2 ounces.

M. Sig. A fluid drachm every four hours.

### OBSTINATE CHRONIC GONORRHEA.

In obstinate chronic cases of gonorrhea the following is a useful mixture, which can be applied by means of the endoscopic tube:—

℞ Iodine Resub.........20 grains.
Eucalyptol........... 2 drachms.
Potass. Iodide....... 2 drachms.
Glycerin. Tannate.... ½ ounce.
Acid. Carbol..........20 grains.
Boro Glyceride q. s. ad 2 ounces.

M. Sig. Apply.

### SEMINAL EMISSIONS.

℞ Peacock's Bromides..2 ounces.

Tinct. Ferri. Chlor... ½ ounce.

Aq. Puræ.............1½ ounces.

M. Sig. One or two teaspoonfuls in water one hour after meals and at bedtime.

